# Genetic Structure and Natal Origins of Immature Hawksbill Turtles (*Eretmochelys imbricata*) in Brazilian Waters

**DOI:** 10.1371/journal.pone.0088746

**Published:** 2014-02-18

**Authors:** Maira C. Proietti, Julia Reisser, Luis Fernando Marins, Clara Rodriguez-Zarate, Maria A. Marcovaldi, Danielle S. Monteiro, Charitha Pattiaratchi, Eduardo R. Secchi

**Affiliations:** 1 Instituto de Oceanografia, Universidade Federal do Rio Grande, Rio Grande, Rio Grande do Sul, Brazil; 2 Oceans Institute and School of Civil, Environmental and Mining Engineering, The University of Western Australia, Perth, Western Australia, Australia; 3 Wealth from Oceans Flagship, Commonwealth Scientific and Industrial Research Organisation, Floreat, Western Australia, Australia; 4 Instituto de Ciências Biológicas, Universidade Federal do Rio Grande, Rio Grande, Rio Grande do Sul, Brazil; 5 School of Biological Sciences, Flinders University, Adelaide, South Australia, Australia; 6 Fundação Pró-Tamar, Praia do Forte, Bahia, Brazil; 7 Núcleo de Educação e Monitoramento Ambiental, Rio Grande, Rio Grande do Sul, Brazil; Institute of Biochemistry and Biology, Germany

## Abstract

Understanding the connections between sea turtle populations is fundamental for their effective conservation. Brazil hosts important hawksbill feeding areas, but few studies have focused on how they connect with nesting populations in the Atlantic. Here, we (1) characterized mitochondrial DNA control region haplotypes of immature hawksbills feeding along the coast of Brazil (five areas ranging from equatorial to temperate latitudes, 157 skin samples), (2) analyzed genetic structure among Atlantic hawksbill feeding populations, and (3) inferred natal origins of hawksbills in Brazilian waters using genetic, oceanographic, and population size information. We report ten haplotypes for the sampled Brazilian sites, most of which were previously observed at other Atlantic feeding grounds and rookeries. Genetic profiles of Brazilian feeding areas were significantly different from those in other regions (Caribbean and Africa), and a significant structure was observed between Brazilian feeding grounds grouped into areas influenced by the South Equatorial/North Brazil Current and those influenced by the Brazil Current. Our genetic analysis estimates that the studied Brazilian feeding aggregations are mostly composed of animals originating from the domestic rookeries Bahia and Pipa, but some contributions from African and Caribbean rookeries were also observed. Oceanographic data corroborated the local origins, but showed higher connection with West Africa and none with the Caribbean. High correlation was observed between origins estimated through genetics/rookery size and oceanographic/rookery size data, demonstrating that ocean currents and population sizes influence haplotype distribution of Brazil's hawksbill populations. The information presented here highlights the importance of national conservation strategies and international cooperation for the recovery of endangered hawksbill turtle populations.

## Introduction

After hatching, sea turtles often present an epipelagic stage characterized by wide dispersal, many times over national boundaries and even oceans [1]. This stage is generally followed by recruitment to coastal areas with adequate conditions for feeding, resting and development [2]. Understanding how populations connect and how animals disperse from rookeries to feeding areas is a challenging task, but is essential for setting priorities and defining management strategies for conservation [3]. In this context, molecular genetic data have been fundamental in obtaining relevant information on interpopulational connectivity, migrations and natal origins of marine turtle feeding populations [4]–[7].

Feeding grounds are generally composed of individuals originating from a mixture of sources, being therefore known as “mixed stocks”. Mitochondrial DNA (mtDNA) haplotype frequencies can be used to determine connections between sea turtles at a feeding ground to their areas of origin (rookeries) by Mixed Stock Analysis (MSA), which uses Markov Chain Monte Carlo (MCMC) sampling to estimate the rookery origins of individuals [8]. “Many-to-many” MSA simultaneously estimates origins and destinations of a meta-population of multiple feeding grounds and rookeries [9], providing a more comprehensive understanding of how areas are linked, and the possible movements that animals undertake. It is usually accepted that these movements are influenced by ocean currents: dispersal at the initial epipelagic phase is thought to be mainly shaped by currents due to the low swimming capability of hatchlings [1], [10]; as animals grow and attain more autonomous movement, it is believed that the influence of currents on migrations weakens, but still occurs [11]. The dispersal of post-breeding sea turtles could in fact reflect their previous drift scenarios as hatchlings, with prevailing ocean currents around nesting areas possibly determining how adult turtles select their foraging sites [12]. Multidisciplinary approaches using oceanographic and genetic information are being increasingly applied to studies of the dispersal and migration patterns of sea turtles (e.g. [7], [13]–[15]).

Understanding migratory pathways and origins of animals at their non-reproductive stages is important for determining how impacts such as direct harvest, habitat degradation, and bycatch in fisheries can be shared by seemingly separate populations that are in fact connected [3]. This is of special significance for the critically endangered [16] hawksbill turtle (*Eretmochelys imbricata*), since the tortoiseshell commerce is still debated by the Convention on International Trade in Endangered Species (CITES) [17]. Due to the uncertainty of how a harvest/other impacts in one area will affect other rookeries and feeding areas, effective conservation and/or sustainable use strategies must be based on the extent of connectivity between hawksbill populations; this type of research is therefore a priority for this highly migratory and endangered species [18].

Important hawksbill nesting and feeding areas exist along the Brazilian coast [19]–[23], but the connections between them are still largely unknown [24]. Here, we decrease this gap by describing 740 bp mtDNA haplotypes of hawksbills from five Brazilian feeding aggregates, assessing genetic structure, and estimating natal origins through many-to-many MSA analysis. In addition, we verify the influence of ocean currents on connectivity, inferring origins by analyzing surface drifter data for the Atlantic.

## Materials and Methods

### Ethics statement

According to Normative Instruction 154/March 2007, all capture, tagging, sampling and transport of biological samples of wild animals for scientific purposes must have approval from Instituto Chico Mendes de Conservação da Biodiversidade (ICMBio) SISBIO committees. This study was approved by the Instituto Chico Mendes de Conservação da Biodiversidade, and conducted under SISBIO licenses #225043, #14122, and #159622. All animal handling was performed by trained personnel, following widely accepted and ethical protocols described in [25]. When capturing live turtles, the following measures were taken to alleviate stress: 1) turtles were kept out of the water for a maximum of ten minutes; 2) work was performed in a shaded area; and 3) animals were released at the same location of capture.

### Sampling and laboratory analyses

**Figure 1:**
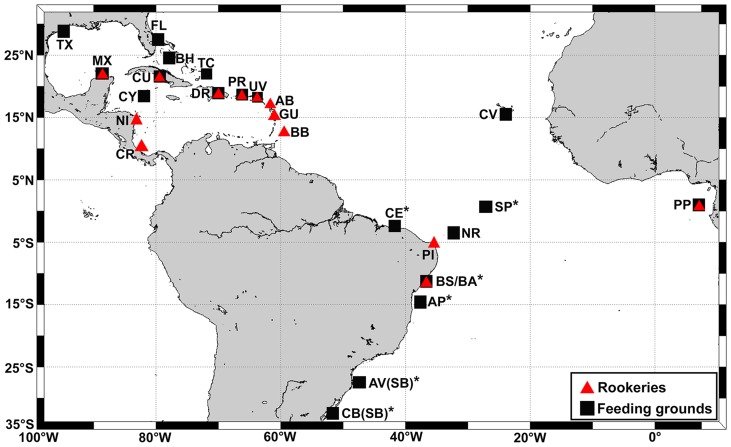
Locations of genetically described hawksbill populations in the Atlantic. Red triangles  =  rookeries, black squares  =  feeding grounds, *study areas described in this work. Rookeries: BS =  Bahia/Sergipe, PI =  Pipa, PP =  Principe, BB =  Barbados, GU =  Guadeloupe, AB =  Antigua & Barbuda, UV =  U.S. Virgin Islands, PR =  Puerto Rico, DR =  Dominican Republic, CR =  Costa Rica, NI =  Nicaragua, CU =  Cuba, MX =  Mexico. Feeding grounds: SP =  São Pedro and São Paulo, NR =  Noronha/Rocas, CE =  Ceará coast, BA =  Bahia coast, AP =  Abrolhos Park, SB =  South Brazil, PP =  Principe, CV =  Cape Verde, UV =  U.S. Virgin Islands, PR =  Puerto Rico, DR =  Dominican Republic, TC =  Turks & Caicos, BH =  Bahamas, FL =  Florida, CU =  Cuba, CY =  Cayman Islands, MX =  Mexico, TX =  Texas.

Skin samples were obtained from 157 immature hawksbills at five areas in Brazilian waters ranging from equatorial to temperate latitudes: (1) São Pedro and São Paulo Archipelago (SPSP, n = 12); (2) Abrolhos National Marine Park (Abrolhos, n = 65), (3) Ceará state coast (Ceará, n = 23); (4) Bahia state coast (Bahia, n = 32); and (5) South Brazil region, which combines the Arvoredo Marine Biological Reserve (n = 6) and Cassino Beach (n = 19). The Arvoredo and Cassino areas were grouped due to small sample size and geographical isolation regarding the other feeding grounds. Two skin samples with approximately 4–5 mm were collected from the fore flippers of each turtle using disposable biopsy punches, and preserved in absolute alcohol. Samples were taken from turtles hand-captured by divers at SPSP, Abrolhos, and Arvoredo, as well as from individuals incidentally caught in fishing nets or stranded on beaches (alive or dead) at Ceará, Bahia, and Cassino Beach. All animals were measured (Curved Carapace Length – CCL), and live turtles were tagged with Inconel Tags (National Tag and Band Co.) using standard techniques [26], [27] prior to release. Sampling locations are shown in Figure 1.

Samples were macerated and kept at 37°C in a lysis buffer until complete digestion. DNA was extracted using Genomic DNA Extraction Kits (Norgen Biotek) or a standard phenol:chloroform method [28]. mtDNA control region fragments of approximately 850 bp were amplified via Polymerase Chain Reaction (PCR) using primers LCM15382/H950 [29], as follows: 5′ at 94°C; 36 cycles of 30″ at 94°C, 30″ at 50°C, 1′ at 72°C; 10′ at 72°C. Samples were purified with Illustra GFX purification kits (GE Healthcare) and sequenced in both directions through capillary electrophoresis using the Applied Biosystems® 3130 Genetic Analyzer (Valid Biotechnology, Universidade Federal de Minas Gerais, Brazil).

### Haplotypes and diversities

Sequences were aligned and cropped to 740 bp using Clustal X 2.0 [30], and classified according to GenBank® and the Atlantic Ocean hawksbill haplotype database (under construction, Abreu-Gobrois pers. comm.). We used 740 bp haplotypes for genetic structure analyses between Brazilian populations, but for comparison with all other Atlantic Ocean feeding grounds and rookeries described in literature, a second cropping to 382 bp (original classification, [31]) was necessary. Arlequin 3.5 [32] was used to assess haplotype (*h*) and nucleotide (*π*) diversities of the study areas.

### Genetic structure

Genetic divergence between feeding populations was verified in Arlequin 3.5 through pairwise fixation indices *F*-st and φ-st, respectively using haplotype frequencies only and a Tamura-Nei model of nucleotide substitution (as determined through jModelTest 0.1.1, [33]). Besides our study areas, the following feeding aggregations were included in genetic structure analyses: (1) Fernando de Noronha/Rocas Atoll, Brazil (referred to as Noronha, n = 94 samples); (2) São Tomé Island, São Tomé and Principe (n = 80); (3) Boavista Island, Cape Verde (n = 28); (4) Buck Island, U.S. Virgin Islands (n = 69); (5) Mona Island, Puerto Rico (n = 256); (6) Dominican Republic (n = 90); (7) Turks and Caicos (n = 38); (8) Bahamas (n = 78); (9) Cuba (n = 210); (10) Cayman Islands (n = 92); (11) Yucatán, Mexico (n = 21); (12) Texas coast, U.S.A. (n = 42); and (13) Florida, U.S.A. (n = 106) ([4]–[7], [24], [34]–[38], for locations see Figure 1). Analysis of Molecular Variance (AMOVA) was implemented to verify ocean basin structure by grouping Atlantic feeding grounds into Brazilian, African, Caribbean and Gulf of Mexico regions. To identify regional structure within Brazilian waters, we divided feeding grounds into two groups: areas influenced mainly by the South Equatorial/North Brazil Current (SPSP, Noronha and Ceará) and those influenced by the Brazil Current (Bahia, Abrolhos and South Brazil).

### Natal origins

A many-to-many MSA using the “mixstock” package in R [9], [39] was performed to estimate source contributions to the study areas in Brazil (feeding ground-centric), as well as the destinations of animals hatched at Brazilian rookeries (rookery-centric). We included all above-cited Atlantic foraging grounds (18 areas, n = 1361 individuals) and thirteen rookeries (n = 875) in this metapopulational analysis: (1) Bahia/Sergipe coasts (referred to as Bahia, n = 92); (2) Pipa Beach (referred to as Pipa, n = 27); (3) Principe Island (n = 20); (4) Barbados (n = 84); (5) Trois Island, Guadeloupe (n = 74); (6) Jumby Bay, Antigua and Barbuda (n = 70); (7) Buck Island, U.S. Virgin Islands (n = 67); (8) Mona Island, Puerto Rico (n = 93); (9) Jaragua and Saona, Dominican Republic (n = 48); (10) Doce Leguas, Cuba (n = 70); (11) Tortuguero, Coast Rica (n = 60); (12) Pearl Cays, Nicaragua (n = 95); (13) Yucatán and Quintana Roo, Mexico (n = 73) ([4], [5], [35], [38], [40]–[44], locations shown in Figure 1). Nesting population size (estimated number of females per nesting season) of each area was included in this analysis as an ecological covariate, following [9]. One MCMC was implemented for each rookery, with chain lengths of 20000, and one half chain discarded as “burn-in”. Gelman-Rubin convergence factors varied from 1.0 to 1.04 (average 1.02), indicating convergence. Hybrid haplotype EixCc BR3, from the Bahia rookery, was maintained in the MSA since it was also observed at feeding areas; orphan haplotypes were excluded.

Lagrangian drifter data was also used to infer on the possible role of ocean currents on the dispersal of hatchlings, following [15]. For such, we downloaded surface drifter data freely available from NOAA's Global Drifter Program (www.aoml.noaa. gov/envids/gld). Areas with size 4°×4° (latitude and longitude) were delineated around all rookeries considered in MSA (n = 13), and the number of drifters that passed through them and reached the Brazilian foraging grounds was counted (drifters with less than three months of transmission were excluded). Feeding areas with similar drifter patterns were grouped into target regions for simplification: 1) SPSP; 2) Noronha and Ceará; and 3) Bahia, Abrolhos and South Brazil. Based on these drifter counts and rookery population size information (females/nesting season), the probability that a turtle arriving at the target region originated from a previously characterized rookery was calculated under a Bayesian framework (for method details refer to [15]).

Finally, natal origin estimates inferred by genetic/rookery size and drifters/rookery size data were compared to better assess the influence of ocean currents on feeding grounds aggregations in Brazil. We evaluated this correlation through a Mantel test implemented with the package “vegan” in R [39], [45], and performed linear regression by regressing log-transformed proportions between genetic and drifter profiles.

## Results

### Haplotypes and diversities

**Figure 2:**
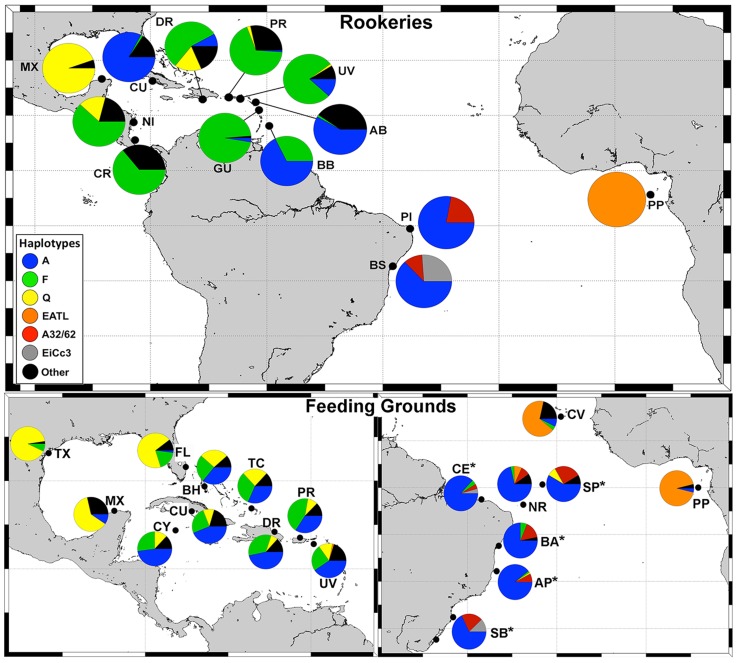
Main haplotype frequencies of genetically described populations in the Atlantic. *study areas described in this work. Rookeries: BS =  Bahia/Sergipe, PI =  Pipa, PP =  Principe, BB =  Barbados, GU =  Guadeloupe, AB =  Antigua & Barbuda, UV =  U.S. Virgin Islands, PR =  Puerto Rico, DR =  Dominican Republic, CR =  Costa Rica, NI =  Nicaragua, CU =  Cuba, MX =  Mexico. Feeding grounds: SP =  São Pedro and São Paulo, NR =  Noronha/Rocas, CE =  Ceará coast, BA =  Bahia coast, AP =  Abrolhos Park, SB =  South Brazil, PP =  Principe, CV =  Cape Verde, UV =  U.S. Virgin Islands, PR =  Puerto Rico, DR =  Dominican Republic, TC =  Turks & Caicos, BH =  Bahamas, FL =  Florida, CY =  Cayman Islands, MX =  Mexico, TX =  Texas.

Haplotype diversities (*h*) of Atlantic feeding areas ranged from 0.143±0.052 at Principe to 0.761±0.035 at Turks and Caicos, with average *h* of 0.538±0.050. Brazilian feeding ground diversity ranged from 0.215±0.052 at Abrolhos to 0.644±0.124 at SPSP; average *h* was 0.418±0.088. We found ten 740 bp haplotypes, with high occurrence (almost 80%) of haplotype A01, followed by a frequency of approximately 8% of A62, and less than 4% for all other haplotypes (Table 1). We found one new haplotype (A92) and four hawksbill x loggerhead (*Caretta caretta*) hybrid sequences (EixCcBR3). When considering only the 382 bp fragment, the number of haplotypes dropped to eight. Short haplotype frequencies (382 bp) of all Atlantic Ocean populations considered in our study can be visualized in Figure 2 and Table S1. Table 2 shows haplotype diversities for all genetically described hawksbill populations in the Atlantic, along with biological information of areas: Curved Carapace Length (CCL) of immature animals at feeding grounds (Loureiro N, Shaver D pers comm, [6], [19], [34], [36], [46]–[52]) and population size of rookeries [17], [22], [23], [44], [53]–[57].

**Table 1:** Haplotype frequencies (740 bp) for Brazilian feeding grounds.

	Brazilian feeding areas				
Haplotypes	SPSP	Ceará coast	Bahia coast	Abrolhos Park	South Brazil
A01/A	7	20	24	58	17
A09/F	0	0	2	1	0
A11/F	0	1	0	0	0
A23/Q	1	0	0	0	0
A24/Q	0	0	0	1	0
A32/f	0	0	3	2	0
A62	3	1	2	3	5
A76	0	0	1	0	0
A92	1	0	0	0	0
EixCcBR3	0	1	0	0	3
n	12	23	32	65	25

Shorter (360 bp) haplotype equivalent is given when available.

SPSP =  São Pedro and São Paulo, South Brazil  =  Arvoredo Reserve/Cassino Beach.

**Table 2:** Haplotype diversities and biological information of Atlantic *E. imbricata* populations.

Feeding areas	N	*h* diversity	CCL range (mean)
**Brazil**			
SPSP	12	0.644±0.128	30–75 (53.7)^a^
Noronha/Rocas (NR)	94	0.516±0.063	26.5–75.5 (52.3)^b^
Ceará coast (CE)	23	0.249±0.116	29–73.2 (37.9)^a^
Bahia coast (BA)	32	0.432±0.113	21–63 (37.7)^a^
Abrolhos Park (AP)	65	0.213±0.056	24.5–58.5 (36.1)^a^
South Brazil (SB)	25	0.434±0.103	30–60 (41)^a^
**West Africa**			
Principe Island (PP)	80	0.143±0.052	18–87 (41.5)^c^
Boavista Island (CV)	28	0.529±0.105	27–62.8 (42.3)^d^
**Caribbean**			
Buck Island (UV)	69	0.757±0.035	27.1–70.5 (43.2)^e^
Mona Island (PR)	256	0.689±0.018	21.5–97* (21.5–32*)^f^
Dominican Republic (DR)	90	0.668±0.033	21–74.4* (26.8–37.4*)^g^
Turks and Caicos (TC)	38	0.761±0.035	19.4–93.0 (41.6)^h^
Bahamas (BH)	78	0.739±0.023	24.3–71.3 (48.8)^i^
Cuba pooled (CU)	210	0.728±0.022	22–66 (35.6)^j^
Grand/Little Cayman (CY)	92	0.687±0.035	20.5–62.6 (32.6)^k^
Rio Lagartos/Yucatán (MX)	21	0.604±0.111	21.5–68.5* (27–48*)^l^
Texas (TX)	42	0.180±0.076	7.0–35 (-)^m^
Florida (FL)	94	0.590±0.049	35.7–83.9(56.6)^n^

Rookeries	N	*h* diversity	N females/season
**Brazil**			
Bahia/Sergipe (BS)	121	0.530±0.043	419* o
Pipa (PI)	27	0.359±0.090	376^p^
**West Africa**			
Principe Island (PP)	20	0.000±0.000	20* q
**Caribbean**			
Barbados (BB)	84	0.441±0.037	504^r^
Trois Island (GU)	74	0.080±0.063	55^s^
Jumby Bay (AB)	52	0.504±0.026	113^t^
Buck Island (UV)	67	0.434±0.070	183^q^
Mona Island (PR)	93	0.600±0.042	340* u
Jaragua/Saona (DR)	48	0.642±0.064	29* v
Doce Leguas (CU)	70	0.213±0.063	563^q^
Tortuguero (CR)	60	0.627±0.059	10^w^
Pearl Cays (NI)	95	0.612±0.044	61^q^
Yucatan (MX)	73	0.363±0.131	668^q^

CCL range is presented for feeding areas; mean number of females per nesting season is given for rookeries.

a This study,

b Bellini 1996,

c Loureiro N (pers comm),

d Monzón-Argüello 2010,

e Boulon 1994,

f van Dam & Diez 1998,

g León & Diez 1999,

h Richardson et al. 2009,

i Bjorndal & Bolten 2010,

j Moncada et al. 2012,

k Blumenthal et al. 2009,

l Garduño-Andrade 2000,

m Shaver D (pers comm),

n Wood et al. 2013,

o Marcovaldi et al. 2007,

p Santos et al. 2013,

q Mortimer & Donnelly 2008,

r Beggs et al. 2007,

s Kamel & Decroix 2009,

t Richardson et al. 2006,

u Diez & van Dam 2012,

v Revuelta et al. 2012,

w Tröeng et al. 2005.

*for feeding grounds: CCL calculated from SCL (SCL =  0.939 * CCL - 0.154, Wabnitz & Pauly 2008); for rookeries: number of females calculated from number of nests (Nfemales  =  Nnests/4).

### Genetic structure

We detected overall significant genetic difference between and within nesting and feeding populations in the Atlantic Ocean (*p*<0.001). *F*-st and φ-st values show that feeding aggregations are not homogenous throughout the Atlantic (Table 3), with significant regional differences: AMOVA confirmed a pronounced structure between Brazilian, African and Caribbean feeding populations (*F*-st = 0.618, *p*<0.001). Structure was also high when dividing feeding areas into Brazil, Africa, Caribbean and Gulf of Mexico groups (*F*-st = 0.553, *p*<0.001). Within Brazilian feeding aggregations, some differences were observed (see Table 3), with two areas being more differentiated from the remainder: the oceanic islands of Fernando de Noronha/Rocas Atoll, off the northeastern coast of Brazil, and the southernmost hawksbill occurrence area, South Brazil. Structure was significant (*F*-st = 0.581, *p*<0.001) between areas under influence of the South Equatorial/North Brazil Current (SPSP, Noronha and Ceará) and those influenced by the Brazil Current (Bahia, Abrolhos and South Brazil).

**Table 3:** Pairwise *F*-st (above diagonal) and φ-st (below diagonal) values between feeding populations in the Atlantic.

	SP	CE	BA	AP	SB	NR	PP	CV	UV	PR	DR	TC	BH	CU	CY	MX	TX	FL
SP	*	**0.024**	**0.017**	**0.055**	**0.025**	**0.013**	0.920	0.753	0.212	0.342	0.167	0.349	0.340	0.233	0.165	0.813	0.900	0.306
CE	**0.023**	*	**0.001**	**0.026**	**0.003**	**0.029**	0.894	0.735	0.233	0.367	0.191	0.337	0.348	0.266	0.190	0.663	0.762	0.339
BA	**0.017**	**0.002**	*	**0.000**	0.087	0.047	0.928	0.815	0.277	0.380	0.218	0.435	0.404	0.279	0.217	0.834	0.891	0.366
AP	**0.054**	**0.026**	**0.001**	*	0.152	0.074	0.944	0.877	0.378	0.437	0.304	0.569	0.503	0.340	0.303	0.902	0.930	0.440
SB	**0.026**	**0.004**	0.089	0.152	*	0.074	0.835	0.642	0.253	0.407	0.238	0.305	0.348	0.318	0.237	0.510	0.621	0.349
NR	**0.013**	**0.030**	0.048	0.074	0.075	*	0.802	0.673	0.230	0.360	0.197	0.307	0.321	0.270	0.196	0.536	0.587	0.383
PP	0.920	0.894	0.929	0.945	0.836	0.803	*	0.068	0.863	0.863	0.874	0.896	0.886	0.856	0.876	0.936	0.950	0.760
CV	0.754	0.736	0.817	0.877	0.643	0.673	0.068	*	0.746	0.802	0.778	0.766	0.788	0.787	0.781	0.817	0.873	0.637
UV	0.211	0.233	0.276	0.376	0.254	0.229	0.867	0.752	*	**0.012**	**0.003**	**0.002**	**0.005**	**0.008**	**0.003**	0.266	0.331	0.127
PR	0.343	0.368	0.380	0.438	0.411	0.362	0.868	0.809	**0.013**	*	0.057	**0.012**	**0.003**	0.058	0.060	0.212	0.244	0.129
DR	0.167	0.192	0.218	0.304	0.241	0.198	0.878	0.784	**0.003**	0.057	*	**0.042**	0.047	**0.010**	**0.009**	0.370	0.428	0.188
TC	0.350	0.336	0.436	0.570	0.305	0.305	0.899	0.771	**0.002**	**0.012**	**0.042**	*	**0.016**	**0.003**	0.044	0.236	0.329	0.070
BH	0.341	0.349	0.405	0.504	0.349	0.320	0.889	0.794	**0.005**	**0.003**	0.048	**0.016**	*	**0.007**	0.047	0.217	0.274	0.087
CU	0.234	0.268	0.279	0.341	0.323	0.271	0.862	0.794	**0.008**	0.048	**0.010**	**0.003**	**0.007**	*	**0.009**	0.266	0.298	0.164
CY	0.166	0.191	0.218	0.303	0.240	0.196	0.880	0.787	**0.003**	0.060	**0.009**	0.044	0.048	**0.009**	*	0.374	0.431	0.190
MX	0.815	0.658	0.835	0.903	0.505	0.530	0.937	0.819	0.265	0.211	0.371	0.236	0.218	0.266	0.375	*	0.053	**0.017**
TX	0.914	0.764	0.899	0.936	0.619	0.583	0.952	0.876	0.331	0.243	0.430	0.334	0.277	0.298	0.434	0.065	*	**0.001**
FL	0.153	0.194	0.206	0.264	0.221	0.255	0.610	0.466	0.066	0.081	0.106	0.028	0.045	0.105	0.107	**0.016**	**0.000**	*

Bold indicates lack of significant difference (p>0.05). SP =  São Pedro and São Paulo, CE =  Ceará coast, BA =  Bahia coast, AP =  Abrolhos Park, SB =  South Brazil, NR =  Noronha/Rocas, PP =  Principe, CV =  Cape Verde, UV =  U.S. Virgin Islands, PR =  Puerto Rico, DR =  Dominican Republic, TC =  Turks & Caicos, BH =  Bahamas, FL =  Florida, CY =  Cayman Islands, MX =  Mexico, TX =  Texas, FL =  Florida.

### Natal origins

**Figure 3:**
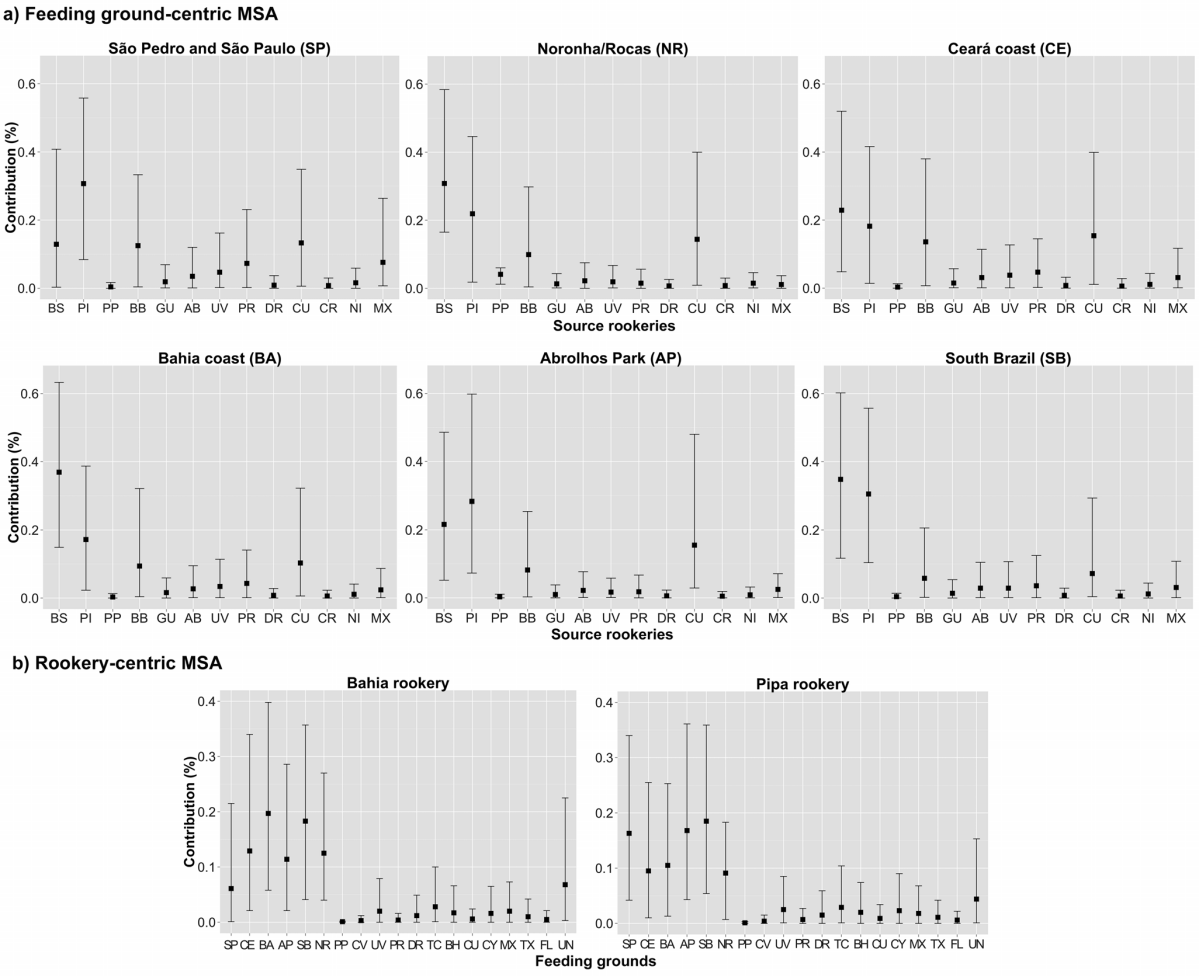
Many-to-many mixed stock analysis estimates for Brazilian populations. (a) shows feeding ground-centric estimates, and (b) rookery-centric estimates. MSA outputs for all other areas are shown in Figures S1 and S2. BS =  Bahia/Sergipe, PI =  Pipa, PP =  Principe, BB =  Barbados, GU =  Guadeloupe, AB =  Antigua & Barbuda, UV =  U.S. Virgin Islands, PR =  Puerto Rico, DR =  Dominican Republic, CR =  Costa Rica, NI =  Nicaragua, CU =  Cuba, MX =  Mexico, SP =  São Pedro and São Paulo, CE =  Ceará coast, BA =  Bahia coast, AP =  Abrolhos Park, SB =  South Brazil, NR =  Noronha/Rocas, PP =  Principe, CV =  Cape Verde, TC =  Turks & Caicos, BH =  Bahamas, FL =  Florida, CY =  Cayman Islands, TX =  Texas, FL =  Florida, UN =  Unknown.

Many-to-many MSA estimates suggest that the feeding aggregations in Brazil are mostly composed of animals originating from domestic rookeries, but also present some distant origins (Figure 3a). The Brazilian rookeries Bahia and Pipa were the predominant sources, with respective contributions of approximately 12% and 28% for SPSP, 30% and 22% for Noronha, 22% and 18% for Ceará, 36% and 17% for Bahia, 21% and 28% for Abrolhos, 34% and 30% for South Brazil. Rookeries from Africa and the Caribbean showed generally lower but noteworthy contributions: Principe, Barbados and Cuba contributed respectively 8%, 10% and 14% to Noronha; Barbados, Puerto Rico and Cuba 12%, 9% and 13% to SPSP; Barbados and Cuba 12% and 15% to Ceará, 9% and 10% to Bahia, 8% and 13% to Abrolhos.

Rookery-centric MSA results for the Brazilian rookeries of Bahia and Pipa shows that these nesting areas contribute mainly to the domestic feeding populations: respectively 6% and 16% to SPSP; 12% and 9% to Ceará; 20% and 10% to Bahia; 11% and 17% to Abrolhos; 18% and 18% to South Brazil; and 10% and 12% to Noronha (Figure 3b). All other contributions were lower than 5%. MSA outputs for overseas feeding grounds and rookeries (not discussed in this study) are presented in Figures S1 and S2.

**Figure 4:**
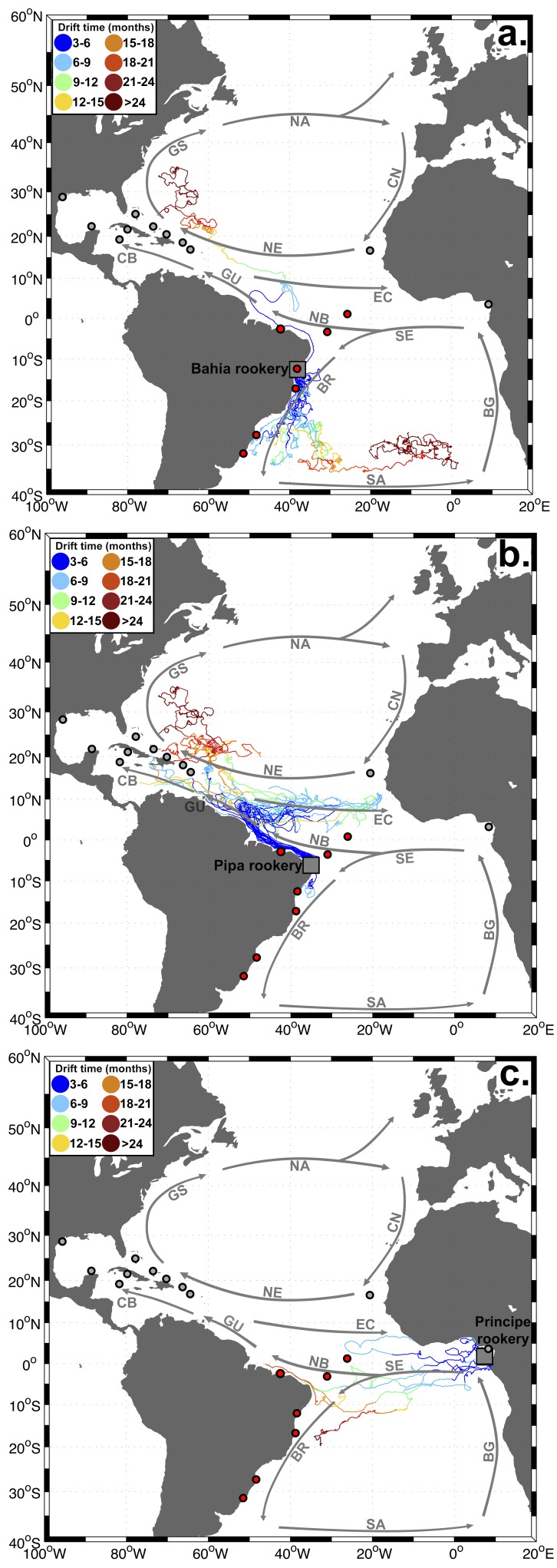
Pathways of drifters passing by Atlantic Ocean rookeries Bahia (a.), Pipa (b.) and Principe (c.). Drifter pathways for the other Atlantic rookeries are shown in Figure S3. Colors indicate drift time (in months). Grey squares  =  drifter release areas around the rookeries, red circles  =  Brazilian feeding grounds, grey circles  =  other Atlantic Ocean feeding grounds. Grey arrows indicate the flow of the major Atlantic Ocean currents: SE =  South Equatorial Current, BR =  Brazil Current, SA =  South Atlantic Current (displaced to the North for illustrative purposes), BG =  Benguela Current, NB =  North Brazil Current, GU =  Guiana Current, CB =  Caribbean Current, EC =  Equatorial Counter Current, NE =  North Equatorial Current, GS =  Gulf Stream, NA =  North Atlantic Current, CN =  Canary Current.

**Figure 5:**
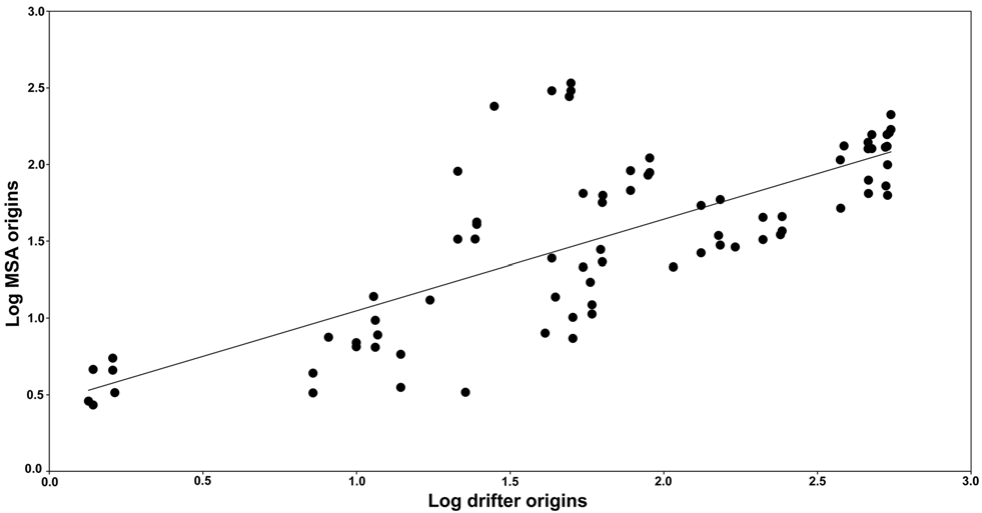
Correlation between genetic and drifter profiles for Brazilian feeding grounds. Genetic profiles are based on log-transformed data of origins from genetic/population size (MSA – x axis) and drifter/population size (y axis) information profiles.

Of the available drifter data, a total of 469 drifters passed through the Atlantic rookeries, of which 388 transmitted for over three months. Of these, 37 drifters arrived at our three target areas in Brazil, originating from the Bahia, Pipa and Principe rookeries (trajectories shown in Figure 4). Displacements from rookeries to Brazilian feeding areas were most likely by means of the North Brazil Current for drifters leaving the Pipa rookery, North Brazil/Brazil Current for those leaving Bahia, and South Equatorial Current for drifters from Principe. No drifters from the Caribbean arrived at the Brazilian coastline (see Figure S3). When estimating natal origins of hawksbills at our target areas through drifter/population size data, significant contributions were: Pipa (58%), Bahia (9%) and Cuba (11%) for SPSP; Pipa (62%), Bahia (14%) and Cuba (10%) for Noronha/Ceará; and Bahia (72%) for Abrolhos/Bahia/South Brazil (Table 4). Correlation between natal origins as calculated by MSA (which included genetic/rookery size data) and drifters/rookery size information was significant (Mantel test, *r* = 0.791, *p*<0.05; linear regression *r* = 0.560, *p*<0.01; Figure 5).

**Table 4:** Natal origins for *E. imbricata* in Brazilian waters as estimated by drifters.

	Target areas		
Rookeries	SPSP	NR/CE	BA/AP/SB
Bahia/Sergipe	0.085	0.139	0.720
Pipa	0.584	0.622	0.072
Principe	0.069	0.023	0.020
Barbados	0.027	0.020	0.017
Guadeloupe	0.003	0.002	0.002
Antigua	0.006	0.005	0.004
USVI	0.009	0.008	0.007
Puerto Rico	0.024	0.018	0.016
Dom Republic	0.003	0.002	0.002
Cuba	0.113	0.100	0.087
Costa Rica	0.003	0.002	0.002
Nicaragua	0.016	0.013	0.011
Mexico	0.058	0.047	0.041

SPSP =  São Pedro and São Paulo Archipelago; NR/CE =  Noronha/Ceará; BA/AP/SB =  Bahia, Abrolhos, South Brazil.

## Discussion

### Haplotypes and diversities

As illustrated in Figure 2, several haplotypes observed at Brazilian feeding areas are common at rookeries located in the Caribbean (A01/A, A09/F, A11/F, A24/Q), Brazil (A32, A62, EixCcBR3) and Africa (EATL). We also detected two orphan haplotypes (i.e. not seen at rookeries): A76 and A92 (the latter previously undescribed); as noted in [35], [58], the presence of orphan haplotypes indicates the need for further sampling of rookeries. Mean haplotype diversity of feeding areas in Brazil was 0.413, which is higher than the mean diversity of the two described feeding grounds off West Africa (*h* = 0.335), but lower than the diversity of ten populations in the Caribbean (*h* = 0.647). These low diversities could be a result of natal origins from fewer, less diverse sources when compared to the Caribbean, where a large number of rookeries with relatively high diversities are present. SPSP and Noronha, located respectively around 350 and 1000 km off northeast Brazil, presented the highest haplotype diversities within the sampled Brazilian feeding grounds. Bass et al. (2006) suggest that feeding areas located at the confluence of several current systems present higher haplotype diversities [59]; Vilaça et al. (2013) propose that Noronha's location, influenced by both the North Brazilian and South Equatorial Currents, is a possible explanation for its elevated diversity [24]. Our results support the hypothesis that ocean currents influence diversity since SPSP, located near Noronha, showed highest diversity values among all Brazilian feeding grounds.

### Genetic structure

Our study suggests regional genetic structuring within the Atlantic, between (i) foraging areas off Brazil, Africa and the Caribbean, a pattern that has been previously noted [24], and (ii) feeding areas in the Gulf of Mexico and the remainder within the Caribbean. Although turtles disperse widely, they can be constrained to certain regions due to factors such as geographical distance, rookery population size, animal behavior and surface ocean currents. For instance, the Gulf of Mexico presents a distinct current pattern (as shown in Figure S3) that could retain animals within the region. In a general manner differentiation between Brazilian feeding grounds was not pronounced, but significant structuring was observed when grouping Brazilian populations into groups influenced by different ocean currents. This suggests that migration patterns are somewhat limited, and that genetic composition could be affected by the different factors noted above. We highlight here the importance of analyzing longer haplotype sequences, which present higher resolution and better detect population structure [40], [60]. Although longer haplotypes are being increasingly analyzed, past hawksbill turtle studies mostly characterize 382 bp fragments; it is important that future studies focus on larger fragments to improve our understanding on structure and connectivity of hawksbill populations.

### Natal origins

Many-to-many MSA showed that feeding aggregations in Brazil are generally composed of animals from limited sources, being mostly linked to the proximal Brazilian rookeries. Results also indicated that in some cases connectivity extends to rookeries located in the Caribbean and West Africa. Origins calculated from drifter data support main contributions from Bahia and Pipa, with almost all drifters arriving from national rookeries to the Brazilian feeding grounds, via the North Brazil and Brazil currents. The only other work to describe origins of hawksbills at a Brazilian feeding ground (Noronha/Rocas) also shows that main origins are from national sources, with lower contributions from other regions [24].

Our rookery-centric MSA, including the five populations investigated in this study plus the Noronha/Rocas feeding ground, decreased contributions of the Bahia rookery to “unknown” feeding grounds from almost 60% (reported in [24]) to less than 10%, and showed strong connections to feeding grounds in national waters. This is likely due to the detection at our feeding areas of a hybrid hawksbill x loggerhead haplotype (EixCc3 BR3), present in 18% of samples from the Bahia rookery [41]. This haplotype had not been registered at feeding grounds sampled by [24], but we report its occurrence at Ceará (4% frequency) and in the temperate waters of South Brazil (16% frequency) [61]. This demonstrates the importance of thorough genetic analyses of populations and the use of metapopulational approaches (ocean basin scale) in animal populations that are demographically connected between distant geographic localities.

Natal origin estimates also showed connection between West Africa and Brazil, with 8% contribution from Principe to Noronha. This is expected since the EATL haplotype, present with 100% frequency at the Principe rookery [35], was encountered at this feeding area. However, drifter tracks actually show a higher connection with West Africa by means of the South Equatorial Current; for example, some drifters at SPSP originated from Principe, but MSA showed no contribution from this rookery to this region as haplotype EATL was not detected. The displacement to the opposite direction via the Equatorial Counter Current is also likely as shown through drifter tracks. Furthermore, hawksbill tag returns have repeatedly evidenced transatlantic movements between these regions [62], [63]. Increasing the number of analyzed samples from SPSP and other feeding grounds along the coast would possibly lead to the detection of the EATL haplotype and further evidence the connection between African and Brazilian hawksbill turtle populations.

MSA showed that Caribbean rookeries contributed to all Brazilian feeding grounds except South Brazil. Highest contributions were observed for SPSP, Noronha, and Ceará, which aggregated animals from up to three Caribbean rookeries. It is hypothesized that as sea turtles grow their feeding habitats become preferentially established near their natal beach [4]. Immature hawksbills sampled at SPSP and Noronha presented larger size classes (Table 2) than those at the other feeding areas in Brazil, possibly indicating that some animals are moving closer to the Caribbean for the onset of reproduction.

The connection between Caribbean and Western South Atlantic green and hawksbill turtle populations has been extensively shown through MSA [13], [15], [24], [64] and tag returns [65]. However, oceanographic data have yet to bring additional evidence to these links. In this study no drifters from Caribbean sources arrived in Brazilian waters, although the opposite displacement occurred. Drifter data are not abundant for every region and the life span of drifters could be too short (∼291–450 d; [66]) to detect this connection. Particle modeling could solve the temporal limitation of drifters, but a study using particle tracking models (dispersal time  =  one year) also did not reveal any virtual drifter from Caribbean hawksbill rookeries arriving around Brazil [7]; a 5-year particle tracking study from the green turtle rookery Costa Rica also did not show this connection [13]. Contribution from the Caribbean to Brazil could be overestimated by MSA due to shared haplotypes between rookeries of these regions; on the other hand, MSA estimates might be correct but animals are undergoing movements undetected by drifters/particle models (e.g. transport by coastal currents in shallow areas or active swimming). Additional genetic characterization of rookeries and feeding areas, as well as further description of animal behavior and oceanographic features on the routes between them, is necessary for resolving this ambiguity.

Although surface drifter patterns did not corroborate MSA origins in all cases, origins calculated by combining drifter data with population size of rookeries (see Table 4) were strongly correlated with MSA estimates. This indicates that ocean currents influence how patterns of hawksbill population genetic structure are shaped in Brazil. Ocean currents apparently play a substantial role in shaping populations in other regions as well; for example, a particle tracking study found significant correlation between particle distribution patterns and natal origin estimates for foraging aggregations in the Caribbean [7]. Restricting MSA to only small turtles, more likely to have recently recruited than larger juveniles that may have already conducted developmental migrations [67], could increase the correlation between natal origins and ocean currents. For allowing this type of meta-analysis, researchers should publish their datasets containing size/haplotype of individual turtles. In light of this suggestion, data from the present work was published in Figshare [68].

The integration of genetic analysis and oceanographic data is a valuable approach to understanding origin and distribution of immature animals; however, there are some caveats inherent to the analyses applied here. Mixed stock analysis may not adequately detect connections between some areas since not all source rookeries are thoroughly characterized, while those that are do not always present highly differentiated haplotype frequencies, leading to high uncertainty [58]. Furthermore, drifter trajectories might not represent the precise pathways of sea turtles, as analyses cannot be limited to hatching seasons due to large reduction of data, and drifters generally have short life spans and do not consider surface wind drag and turtle behavior/swimming [66]. Despite these caveats, the type of information described above has proven to be quite useful for indicating possible movements and migrations between areas (e.g. [10], [15], [69], [70]). Ocean circulation models are also being increasingly used, and allow choosing the number and lifespan of particles, release periods, and incorporating additional factors such as wind drag and swimming power of hatchlings [71], [72]. However, they are also limited due to uncertainties associated with simulation of turtle behavior and spatio-temporal resolution unable to capture fine-scale features [73]. An ideal scenario would integrate several connectivity indicators; for achieving this, other means of linking populations should continue to be investigated. Stable isotope analysis, for example, is currently being used to distinguish populations and unveil migratory origins by comparing isotopic signatures of animals to predominant regional isoscapes [74]–[76].

### Implications for hawksbill conservation

How populations connect, and how impacts on any particular population will affect others, are fundamental questions for the conservation of endangered, highly migratory animals. Hawksbill turtles are currently listed as critically endangered in the IUCN red list [16], and listed on Appendix I of the Convention on the International Trade in Endangered Species. Nevertheless, hawksbill products continue to be commercially valuable and in demand in some regions, and debate has arisen as to whether or not harvesting should be permitted, and at what levels such harvest would be sustainable [4], [77]–[79]. Some studies have tackled these issues and concluded, for example, that exploiting turtles in Caribbean feeding areas would affect rookeries throughout the entire region [4], [79].

Although Brazilian populations have also been historically depleted due to harvest for consumption and fisheries bycatch, nesting populations are currently showing encouraging increases [22], [23]. Our analyses indicate that mixed stocks are composed mostly of animals that originate from Brazilian rookeries, which is a fortunate scenario since a large sea turtle conservation project (Project Tamar-ICMBio) has been underway in Brazil since 1980 [80] and the need for transnational conservation policies is somewhat reduced. Despite this encouraging outlook, it is not yet known if immature feeding population numbers are also rising, and impacts such as bycatch [81] and ingestion of/entanglement in plastic debris [82] are increasingly causing sea turtle mortality. Furthermore, we demonstrate that some areas are demographically and genetically connected to rookeries in the Caribbean and West Africa. For instance, the SPSP and Noronha feeding grounds aggregate large animals from various national and international origins, and impacts at these developmental areas could affect several nesting populations throughout the Atlantic. Although Fernando de Noronha, Rocas Atoll and Arvoredo Reserve are protected areas where fishing is not allowed, other areas, including the waters around the São Pedro and São Paulo Archipelago, are important fishing grounds [83]. The Ceará, Bahia and Cassino Beach areas are also not integrally protected, and impacts at these areas will most likely influence the Brazilian nesting populations, and possibly some Caribbean rookeries.

In this study we reduce the gap in genetic information of hawksbill turtle feeding populations in Brazil, and illustrate how these populations are connected with proximal and distant rookeries in the Atlantic Ocean. The results presented here should be considered in future national and multinational strategies for mitigating bycatch/other impacts to increase protection and recovery of endangered populations of hawksbill turtles.

## Supporting Information

Figure S1 **Feeding ground-centric many-to-many MSA estimates for Atlantic feeding populations.** BS =  Bahia/Sergipe, PI =  Pipa, PP =  Principe, BB =  Barbados, GU =  Guadeloupe, AB =  Antigua & Barbuda, UV =  U.S. Virgin Islands, PR =  Puerto Rico, DR =  Dominican Republic, CU =  Cuba, CR =  Costa Rica, NI =  Nicaragua, MX =  Mexico.
(TIFF)

Figure S2 **Rookery-centric many-to-many MSA estimates for Atlantic nesting populations.** SP =  São Pedro and São Paulo, CE =  Ceará coast, BA =  Bahia coast, AP =  Abrolhos Park, SB =  South Brazil, NR =  Noronha/Rocas, PP =  Principe, CV =  Cape Verde, UV =  U.S. Virgin Islands, PR =  Puerto Rico, DR =  Dominican Republic, TC =  Turks & Caicos, BH =  Bahamas, CU =  Cuba, CY =  Cayman Islands, MX =  Mexico, TX =  Texas, FL =  Florida, UN =  Unknown.
(TIF)

Figure S3 **Pathways of drifters passing by Atlantic Ocean rookeries.** Colors indicate drift time (in months). Grey squares  =  drifter release areas around the rookeries, red circles  =  Brazilian feeding grounds, grey circles  =  other Atlantic Ocean feeding grounds. Grey arrows indicate the flow of the major Atlantic Ocean currents: SE =  South Equatorial Current, BR =  Brazil Current, SA =  South Atlantic Current (displaced to the North for illustrative purposes), BG =  Benguela Current, NB =  North Brazil Current, GU =  Guiana Current, CB =  Caribbean Current, EC =  Equatorial Counter Current, NE =  North Equatorial Current, GS =  Gulf Stream, NA =  North Atlantic Current, CN =  Canary Current.
(TIF)

Table S1 **Short haplotype frequencies for Atlantic Ocean hawksbill populations.** Longer haplotype (740 bp) names are given when they have no short equivalent. References for genetic data can be found in the text.
(DOCX)
