# Supplementary figures and images for: Genetic Structure and Natal Origins of Immature Hawksbill Turtles (Eretmochelys imbricata) in Brazilian Waters
Source: PLoS One. 2014 Feb 18;9(2):e88746. doi: 10.1371/journal.pone.0088746 (PMC3928279; doi:10.1371/journal.pone.0088746)

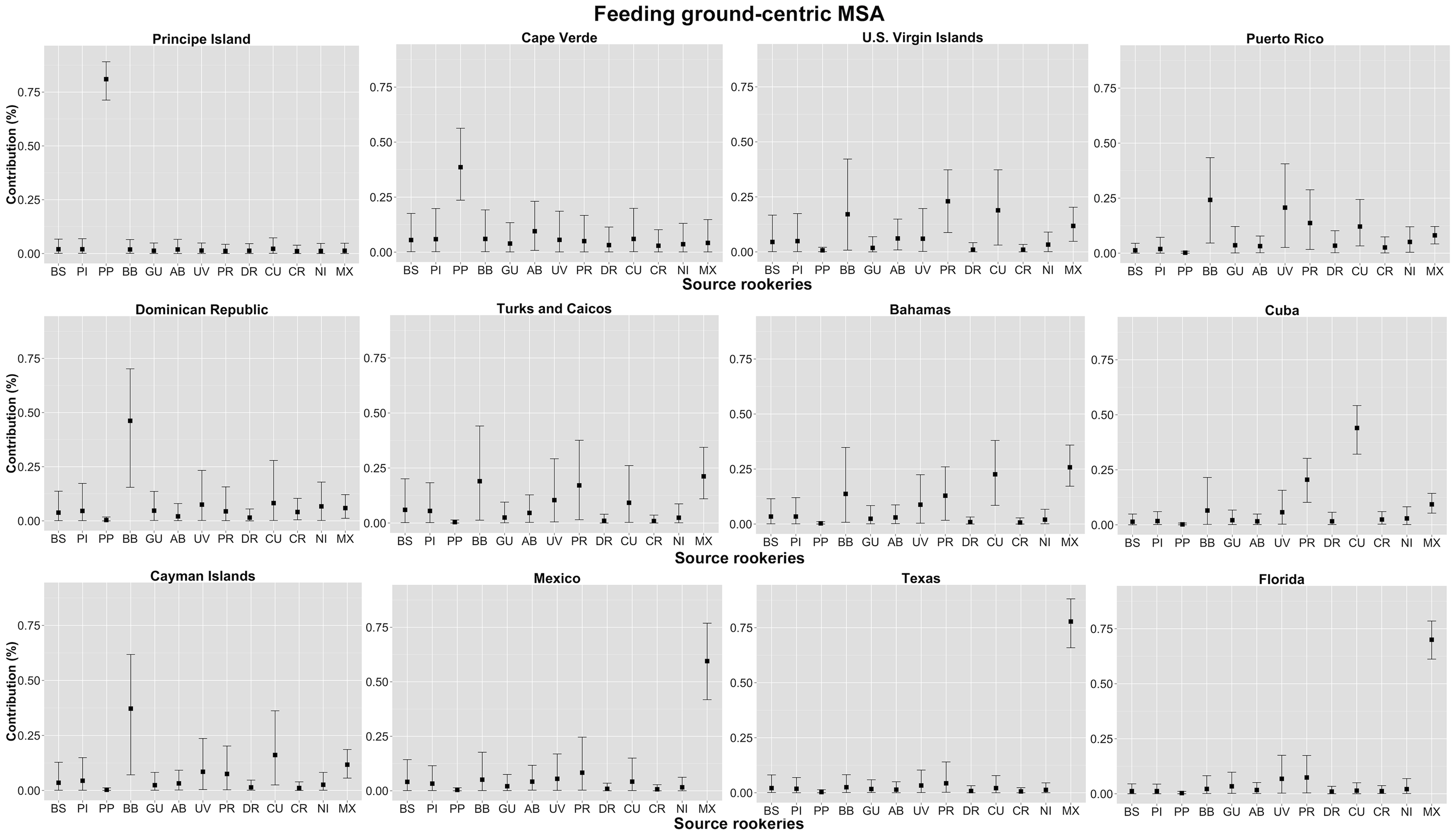

Supplement: Figure S1 — Feeding ground-centric many-to-many MSA estimates for Atlantic feeding populations. BS = Bahia/Sergipe, PI = Pipa, PP = Principe, BB = Barbados, GU = Guadeloupe, AB = Antigua & Barbuda, UV = U.S. Virgin Islands, PR = Puerto Rico, DR = Dominican Republic, CU = Cuba, CR = Costa Rica, NI = Nicaragua, MX = Mexico. (TIFF) [file pone.0088746.s001.tif]

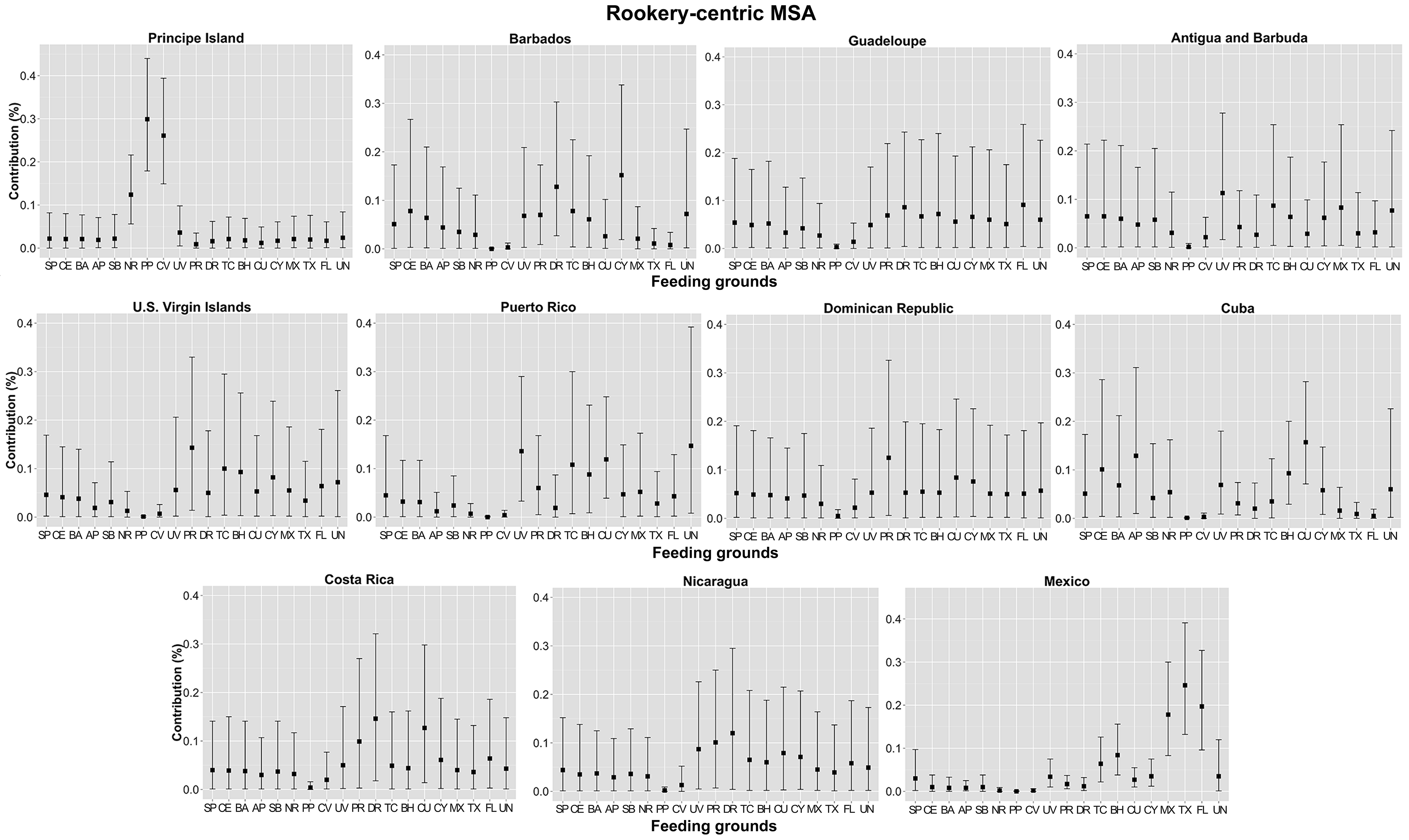

Supplement: Figure S2 — Rookery-centric many-to-many MSA estimates for Atlantic nesting populations. SP = São Pedro and São Paulo, CE = Ceará coast, BA = Bahia coast, AP = Abrolhos Park, SB = South Brazil, NR = Noronha/Rocas, PP = Principe, CV = Cape Verde, UV = U.S. Virgin Islands, PR = Puerto Rico, DR = Dominican Republic, TC = Turks & Caicos, BH = Bahamas, CU = Cuba, CY = Cayman Islands, MX = Mexico, TX = Texas, FL = Florida, UN = Unknown. (TIF) [file pone.0088746.s002.tif]

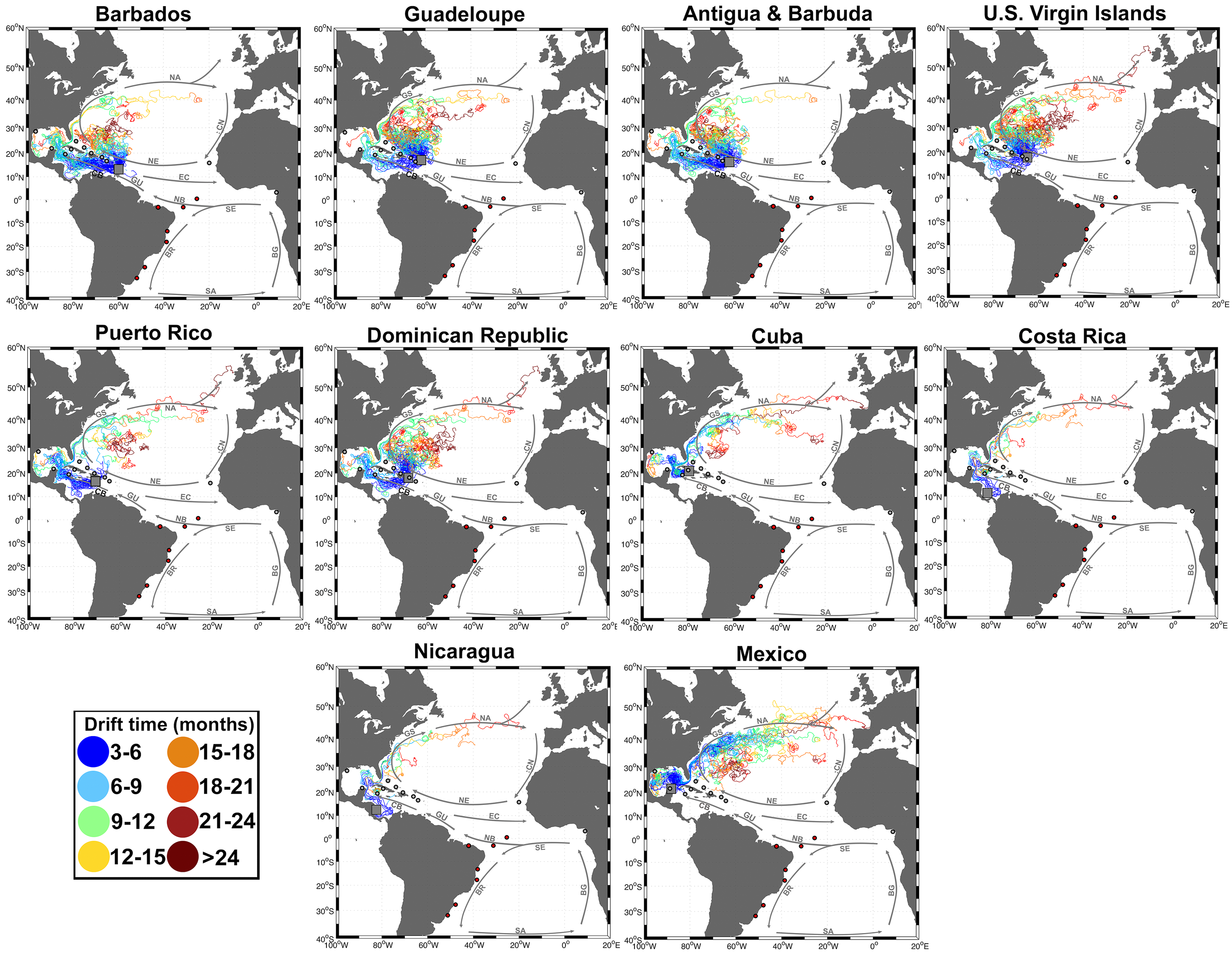

Supplement: Figure S3 — Pathways of drifters passing by Atlantic Ocean rookeries. Colors indicate drift time (in months). Grey squares = drifter release areas around the rookeries, red circles = Brazilian feeding grounds, grey circles = other Atlantic Ocean feeding grounds. Grey arrows indicate the flow of the major Atlantic Ocean currents: SE = South Equatorial Current, BR = Brazil Current, SA = South Atlantic Current (displaced to the North for illustrative purposes), BG = Benguela Current, NB = North Brazil Current, GU = Guiana Current, CB = Caribbean Current, EC = Equatorial Counter Current, NE = North Equatorial Current, GS = Gulf Stream, NA = North Atlantic Current, CN = Canary Current. (TIF) [file pone.0088746.s003.tif]
